# Older Children with Torso Trauma Could Be Managed by Adult Trauma Surgeons in Collaboration with Pediatric Surgeons

**DOI:** 10.3390/children9030444

**Published:** 2022-03-21

**Authors:** Hsiang-Chieh Huang, Tzu-Chi Teng, Yung-Ching Ming, Jainn-Jim Lin, Chien-Hung Liao, Chi-Hsun Hsieh, Pei-Hua Li, Chih-Yuan Fu

**Affiliations:** 1 Department of Pediatric Surgery, Chang Gung Memorial Hospital, Linkou Medical Center, Chang Gung University, Taoyuan City 333, Taiwan; jj4925000@cgmh.org.tw (H.-C.H.); minyc@cgmh.org.tw (Y.-C.M.); 2 Department of Surgery, Chang Gung Memorial Hospital, Linkou Medical Center, Chang Gung University, Taoyuan City 333, Taiwan; jasoncgmh@cgmh.org.tw; 3 Department of Pediatrics, Chang Gung Memorial Hospital, Linkou Medical Center, Chang Gung University, Taoyuan City 333, Taiwan; lin0227@cgmh.org.tw; 4 Department of Trauma and Emergency Surgery, Chang Gung Memorial Hospital, Linkou Medical Center, Chang Gung University, Taoyuan City 333, Taiwan; m7077@cgmh.org.tw (C.-H.L.); hsiehchihsun@yahoo.com.tw (C.-H.H.)

**Keywords:** pediatric torso trauma, trauma surgeon, pediatric surgeon, pediatric critical care

## Abstract

Background: The purpose of this study is to assess the roles of pediatric surgeons and adult trauma surgeons in the management of pediatric torso trauma patients in a Level I adult trauma center. Methods: From 2015 to 2019, pediatric torso trauma patients (age < 18 years) were studied. A comparison between patients who did and did not undergo surgery was performed. Older children (age: 10–18 years) were compared with young adults (age: 18–35 years) selected with the same criteria using propensity score matching (PSM) and inverse probability of treatment weighting (IPTW). Results: A total of 226 patients were included in the study. Patients who underwent surgery for torso trauma (*N* = 61) were significantly older than patients who did not undergo surgery (*N* = 165) (13.1 vs. 10.4 years, *p* = 0.019). Both PSM and IPTW showed that the older children and young adult groups had similar proportions of patients requiring surgery (32.6% vs. 32.6%, standard difference (SD) = 0.000), proportions of patients who required torso angioembolization (8.7% vs. 9.8%, SD = 0.072), length of hospital stay (LOS) (8.1 vs. 8.0 days, SD = 0.026), and intensive care unit admission LOS (2.6 vs. 2.7 days, SD = 0.033). However, 7.1% of older children received critical care from pediatric surgeons. Additionally, 31.9% of younger children were cared for by pediatric surgeons/pediatricians. Conclusions: Adult trauma surgeons can feasibly perform surgeries for older children with torso trauma in collaboration with pediatric surgeons who provide critical care.

## 1. Introduction

Injury is a leading cause of death and disability in children [1]. Injury-related disabilities in children result in reductions in life span and economic productivity [2,3]. Logically, pediatric surgeons are the most suitably equipped for treating pediatric trauma patients. The ideal setting is one in which a pediatric surgeon takes the lead, such as the few trauma centers designed specifically for children. However, the care of injured children has received less attention from the medical field than has the care of adults because the number of pediatric trauma patients is far smaller than the number of injured adults [4]. In addition, the shrinking population and the uneven geographic distribution of pediatric surgeons remain global issues [5,6]. Most trauma centers staff in-house adult trauma surgeons around the clock, while pediatric surgeons are on call and are only contacted if needed. In other words, adult trauma surgeons may need to be involved in the primary management of pediatric trauma patients to ensure timely treatment.

In the current study, we evaluated whether adult trauma surgeons could feasibly treat pediatric torso trauma patients in a Level I adult trauma center. We hypothesized that adult trauma surgeons could provide an acceptable quality of treatment to pediatric torso trauma patients who have specific characteristics. The decision regarding whether a patient should be treated in a pediatric or adult unit was also discussed.

## 2. Methods

### 2.1. Study Setting

From January 2015 to December 2019, pediatric patients (age < 18 years) with torso trauma were studied retrospectively based on our trauma registry and medical records. The definitions of torso trauma (ICD-9-CM: 860.xx–869.xx for chest trauma or abdominal trauma) are shown in Electronic Supplementary Material, Table S1. Patients with severe head injury, abbreviated injury scale (AIS) of the head ≥ 3, patients who died in the emergency department (ED) without further interventions, and patients who were discharged from the ED without admission or operation were excluded [7]. The general demographics (age, sex, body weight, and body height), vital signs, trauma scores reflecting injury severities (AIS for each body region and injury severity score (ISS) for the overall evaluation), details of treatment, and outcomes were routinely recorded and evaluated.

In Taiwan, there are no trauma centers that are specifically designed for pediatric trauma patients. Thus, both adult and pediatric trauma patients are evaluated by adult trauma surgeons. For pediatric patients with torso trauma who required surgical treatment, the surgeries were performed primarily by in-house adult trauma surgeons. On-call pediatric surgeons were contacted during or after the surgeries, if needed. In some patients who did not require surgical interventions, transcatheter arterial embolization (TAE) was used as an adjunct to nonoperative management. After primary treatment, further critical care of pediatric torso trauma patients was provided in the pediatric unit (by both pediatricians and pediatric surgeons) or in the adult unit, a decision made based on clinical judgment.

### 2.2. Study Design

**Figure 1:**
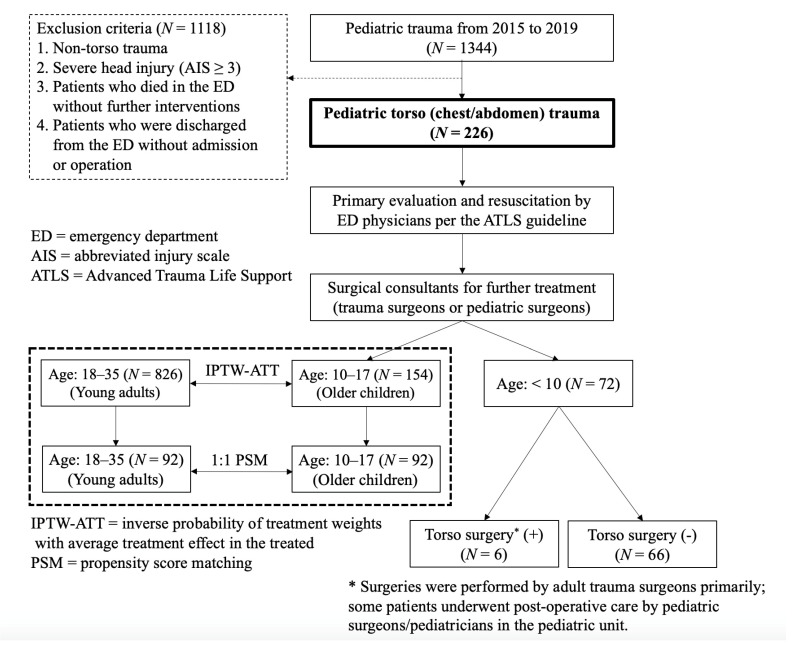
Patient distribution and study protocol of the current study.

The characteristics of the pediatric torso trauma patients in the current study and comparisons between patients who did or did not undergo surgical treatment are shown in Table 1. Patients were divided into two groups (age 10–17 years and age < 10 years) for analyses because these groups had different trauma epidemiologies, physiological characteristics, and policies for treatment (Figure 1) [8,9]. The roles of physicians in treating these two groups of patients were evaluated separately. The surgical indications (unstable hemodynamics with intra-abdominal hemorrhage, hollow viscus injury, massive hemothorax, complicated or deep perineal laceration, uncertain diagnosis, or isolated intra-abdominal free fluid) and associated procedures for each group are listed in Electronic Supplementary Material, Table S2.

For older children (age: 10–17 years), the policy of treatment is presumed to be the same as that for young adults (age: 18–35 years) because they have similar physiological characteristics [10,11]. Therefore, we hypothesized that adult trauma surgeons could provide an acceptable quality of treatment to older children. To prove this hypothesis, the same selection criteria were applied to young adults to select a control group for matching and comparison. We used two analytical approaches, propensity score matching (PSM) and inverse probability of treatment weighting (IPTW) via the average treatment effect in the treated (ATT) patients to minimize selection bias between the older children and young adults. After matching and adjustment were confirmed to yield well-balanced cohorts, treatment strategies—the proportions of patients who required TAE and surgery—and outcomes—hospital length of stay (LOS) and intensive care unit (ICU) LOS—were compared between older children and young adults (Table 2 and Table 3). The mortality rate was too low to be statistically analyzed.

For younger children (age < 10 years), who have completely different physiological characteristics than those of older children and adults, we reported the probability of the need for surgical treatment. The distribution of pediatric torso trauma patients who underwent surgery across age groups (Electronic Supplementary Material, Figure S1) and the characteristics of younger children whose torso trauma surgery was performed by adult trauma surgeons are provided in Electronic Supplementary Material, Table S3.

Finally, the proportions of patients who were treated in the pediatric unit by pediatric surgeons/pediatricians and the proportion of patients who were treated in the adult unit were presented and compared between older and younger children (Table 4).

### 2.3. Statistical Analysis

In the current study, both older children and young adults were defined according to the World Health Organization definitions (adolescents were considered older children) [12]. Nominal data are presented as numbers with percentages and were compared using chi-square tests, and numerical data are presented as the mean ± standard deviation and were compared using Student’s t-tests. A value of *p* < 0.05 was considered statistically significant.

A one-to-one PSM methodology was used to construct pairs between older children and young adults using the greedy neighbor approach [13,14]. A caliper setting of 0.1 was utilized (Table 2). Furthermore, IPTW with ATT was used to avoid excluding several hundred patients from PSM [15]. Each patient was assigned an inverse weighting of the young adults or older children using calculated propensity scores and the following formula: young adult = 1, older children = propensity score/1-propensity score. Therefore, the weight of young adults was decreased, while the weight of older children was increased. This method made these two groups as comparable as possible (Table 3).

The standardized difference (SD) was used to confirm a balanced matching result. The matching result was considered balanced when the SD was less than 0.1 [16].

## 3. Results

During the 48-month study period, 226 pediatric patients with torso trauma were included. The average age was 11.1 years, and the sex ratio (male/female) was 2.5. These patients had an average body height of 145.4 cm and an average body weight of 40.0 kg. There were 154 (68.1%) older children (age: 10–17) and 72 (31.9%) younger children (age: < 10). The overall mortality rate was 2.2% (*N* = 5, 11-year-old female with pelvic fracture-related uncontrolled bleeding, 9-year-old male with liver injury-related uncontrolled bleeding, 10-year-old male with sepsis-related multiple organ failure, 15-year-old male with sepsis-related multiple organ failure, and 12-year-old male with sepsis-related multiple organ failure), and the average hospital LOS and ICU LOS were 7.1 days and 2.8 days, respectively. The patient distribution and study protocol of the current study are shown in Figure 1.

In the current study, 61 pediatric torso trauma patients (27.0%, 61/226) underwent surgical treatment performed by adult surgeons. Compared with the patients who did not undergo surgery (*N* = 165), those who underwent surgery for torso trauma were significantly older (13.1 vs. 10.4, *p* = 0.019), taller (159.4 vs. 140.2 cm, *p* = 0.022), and heavier (58.8 vs. 33.0 kg, *p* < 0.001). There were significantly more older children who underwent surgical treatment than those who did not undergo surgical treatment (90.2% vs. 60.0%, *p* < 0.001) (Table 1).

Of all the pediatric torso trauma patients (*N* = 226), 154 were older children (age: 10–17 years). The same selection criteria were used to identify 826 young adults (age: 18–35 years). Before matching, the older children (*N* = 154) and young adults (*N* = 826) had significantly different demographics, vital signs, and injury severities (left side of Table 2). PSM yielded a well-balanced cohort of 184 patients from these 154 older children and 826 young adults. After matching, the groups of older children and young adults had similar proportions of patients who required surgery (32.6% vs. 32.6%, SD = 0.000) and torso TAE (8.7% vs. 9.8%, SD = 0.072). In addition, there was no significant difference in hospital LOS (8.1 vs. 8.0 days, SD = 0.026) or ICU LOS (2.6 vs. 2.7 days, SD = 0.033) between the older children and young adults after matching (right side of Table 2). In addition to PSM, the adjustment after IPTW showed that the treatment policies (surgery: 33.1% vs. 32.8%, SD = 0.008; torso TAE: 12.1% vs. 13.4%, SD = 0.065) and outcomes (hospital LOS: 11.4 vs. 12.0 days, SD = 0.093; ICU LOS: 3.6 vs. 3.7 days, SD = 0.050) were similar between older children and young adults (Table 3).

Although surgery for older children could be performed by adult trauma surgeons, in the current study, 7.1% of older children received treatment in the pediatric unit from pediatric surgeons/pediatricians. Compared with older children, younger children had a significantly higher proportion of patients who were treated in the pediatric unit (3.9% vs. 7.1%, *p* < 0.001) (Table 4). Among the younger children (*N* = 72), only six patients (8.3%) underwent surgical treatment for torso trauma (Electronic Supplementary Material, Figure S1). One 6-year-old girl underwent a diversion colostomy for open pelvic fracture (body height = 114 cm, body weight = 19 kg). One 7-year-old boy underwent splenectomy because of a high-grade splenic laceration with unstable hemodynamics (body height = 123 cm, body weight = 25.2 kg). The other four younger children who underwent surgeries were 131 cm/40 kg, 138 cm/46 kg, 140 cm/48 kg, and 152 cm/61 kg, respectively, and their body sizes were similar to those of adults (Electronic Supplementary Material, Table S3).

## 4. Discussion

### 4.1. Role of Adult Trauma Surgeons in the Management of Pediatric Torso Trauma Patients

Some studies have compared the quality of care provided to pediatric trauma patients among institutions. Rhodes et al. found equivalent mortality rates and no difference in the incidence of preventable deaths among pediatric trauma patients treated at Level I adult trauma centers [10]. Osler et al. showed no significant difference in injury severity-adjusted survival between children treated at pediatric and adult trauma centers [17]. Others have demonstrated equivalent mortality rates between pediatric patients treated by pediatric surgeons and adult surgeons [18,19,20,21,22]. On the other hand, Potoka et al. reported a significantly lower mortality rate in trauma centers with pediatric trauma care programs [23]. Densmore et al. found that in-hospital mortality, LOS, and costs were all significantly higher when seriously injured pediatric patients were treated in adult hospitals than when they were treated in children’s hospitals [24]. According to these previous publications, it is unclear whether pediatric trauma patients should be managed by pediatric surgeons.

Regardless of the presence of head injury, which is the predominant form of trauma in younger children, most torso trauma in younger children can be managed conservatively [25,26,27] The elastic rib cage and increased thickness of the organ capsule in these patients may contribute to the high success rate of the nonoperative management of torso trauma [4,28,29,30]. In the current study, patients who underwent surgery were significantly older than patients who did not. In other words, compared with younger children, older children have a higher probability of requiring surgery for torso trauma because they are physically more similar to adults. It was reported that the difference between older children and adults was mostly psychological rather than physiological [10]. Older children and adults may have similar physiological and anatomical characteristics [10,11]. Therefore, logically, surgeries for pediatric torso trauma can be performed by adult trauma surgeons.

Table 1 shows that over 90% (90.2%) of patients who underwent surgical treatment were older children whose physiological characteristics were similar to those of adults. This result supports our hypothesis. Adult trauma surgeons play a role in the management of pediatric torso trauma. Moreover, we evaluated whether the treatment strategies were the same between older children and young adults with similar physiological characteristics. The quality of treatment should also be considered when surgeries for older children are performed by adult trauma surgeons. After PSM and IPTW yielded well-balanced cohorts, there was no significant difference in the need for surgery (surgical procedures or angioembolization) between older children and young adults in the current study. The outcomes (ICU LOS and hospital LOS) were also remarkably similar between these two groups of patients (Table 2 and Table 3). These results indicate that the policy of torso trauma management was the same for young adults and older children and that adult trauma surgeons can provide equally good care to both of these groups of patients. Hence, adult trauma surgeons could feasibly perform surgeries in pediatric torso trauma patients.

### 4.2. Role of Pediatricians/Pediatric Surgeons in the Treatment of Pediatric Torso Trauma Patients

Another open issue is the role of pediatricians/pediatric surgeons in the management of pediatric trauma patients. Generally, children cannot be evaluated and treated as if they are smaller versions of adults [31]. Due to their physiological differences, children are able to more effectively compensate for hemorrhage, sustain different injuries from the same injury mechanism, and present management challenges resulting from their smaller size and other anatomical differences [32]. Although older children, who are similar to adults, can be managed by adult trauma surgeons with acceptable outcomes, we believe that pediatricians and pediatric surgeons play a key role in their critical care. Furthermore, younger children have unique physiological and anatomical characteristics from those of older children. The specific care provided by pediatricians or pediatric surgeons is vital for such patients, who have completely different normal ranges of vital signs and laboratory test results. In addition, the dose of medicine, amount of fluid resuscitation, blood transfusion, and even phlebotomy access in younger children require the specific experience and techniques available in pediatric units. Moreover, adult trauma surgeons are usually not familiar with communicating with children and parents. Sathya et al. found that younger children (≤5 years old) had a higher risk of dying when treated at an adult trauma center, but there was no association between the type of trauma center and the risk of death in older children (6–11 years old) or adolescents (12–18 years old) [33].

Practically, pediatric surgeons are usually needed for pediatric patients who are extremely young (e.g., age < 5 years old or neonatal). The role of pediatric surgeons is important not only for technical reasons, such as the repair of small organ injuries (small bowel, ureter, etc.) but also because of socioeconomical support from the pediatric department. Especially for cases of domestic violence, collaboration and partnership between pediatric surgeons/pediatricians and social workers are also advantageous for children. In our institution, approximately 3–5 pediatric torso trauma patients per year may require assistance from pediatric surgeons during operations, which are otherwise primarily performed by adult trauma surgeons. Some patients underwent surgery performed by adult trauma surgeons but were postoperatively treated by pediatric surgeons/pediatricians.

In the current study, pediatric trauma patients were admitted to either the pediatric unit or adult unit based on clinical judgment in our institution. We observed that patients who were treated in the pediatric unit were significantly younger than those who were treated in the adult trauma unit. This reflects a lack of confidence in the management of younger children by adult trauma surgeons, whereas such work is easy for pediatricians or pediatric surgeons. A collaboration between adult trauma surgeons and pediatric surgeons in the management of pediatric torso trauma patients could reduce the burden on pediatric surgeons and serve as an alternative strategy for address the shrinking population of pediatric surgeons.

### 4.3. Boundary between Children and Adults

The definition of a pediatric trauma patient is most commonly based on age, and physicians who treat these pediatric patients can be defined accordingly. However, the cutoff age between childhood and adulthood is still controversial. The National Pediatric Trauma Registry includes patients up to the age of 21 years [34,35]. Other studies have used upper age limits for pediatric trauma patients ranging from 15 to 20 years [18,21,24,36,37,38]. The Pennsylvania Trauma Systems Foundation settled on 14 years as the upper limit for pediatric patients [39]. Others have suggested that puberty is the cutoff for the categorization of pediatric trauma patients [10]. In addition, some patients who are classified as children may have a body size similar to that of an adult because of their nutrition status. This fact contributes to the rationale for the provision of care to older children by adult trauma surgeons. In the current study, most pediatric patients who underwent surgery were 17 years old. In fact, 17-year-old patients and 18-year-old patients were almost the same; the treatment strategies and outcomes should be the same in these two groups of patients. There should be no difference in the treatment provided by pediatric surgeons and adult trauma surgeons.

Although there were six patients under 10 years old who underwent surgeries performed by adult trauma surgeons, most of these patients had body sizes comparable to those of young adults. Therefore, the determination of whether adult or pediatric surgeons should treat trauma patients using a single number (age) is arbitrary; body height and weight should also be considered.

## 5. Limitations

Several limitations of this study need to be considered. First, our results were obtained from a single institution, and the results may not be generalizable because of the varying numbers of trauma centers, their accessibility, and the training and competence of trauma providers in other systems. Second, there are no defined criteria for admitting patients to either the adult care unit or pediatric care unit in our institution. The decision made by emergency medical services regarding admission could have potentially been biased by undocumented factors. Third, there were no outcomes for patients who underwent surgery performed by pediatric surgeons as a control group. Outcomes and therapies were also not compared between patients who were treated in the adult care unit and the pediatric care unit. Finally, more detailed information representative of the care process could not be accessed because this was a retrospective database study. Further prospective studies with larger sample sizes are required.

## 6. Conclusions

Adult trauma surgeons can feasibly perform surgeries for older children with torso trauma in collaboration with pediatric surgeons who provide critical care. Age is not the only variable that should be considered when determining which physicians should provide treatment.

## Figures and Tables

**Table 1:** Comparisons of characteristics between pediatric torso trauma patients who did and did not undergo surgery (*N* = 226).

Variables	Torso Surgery (+) (*N* = 61)	Torso Surgery (−) (*N* = 165)	*p*-Value
Age (years)	13.1 ± 6.5	10.4 ± 4.1	0.019 *
Older children (N)	55 (90.2%)	99 (60.0%)	<0.001 ^†^
Male (N)	42 (68.8%)	119 (72.1%)	0.632 ^†^
Body height (cm)	159.4 ± 82.1	140.2 ± 50.5	0.022 *
Body weight (kg)	58.8 ± 21.4	33.0 ± 19.9	<0.001 *
SBP in ED (mmHg)	114.2 ± 57.6	120.3 ± 33.4	0.238 *
Pulse in ED (/minute)	110.5 ± 62.2	113.7 ± 64.7	0.722 *
RR in ED (/minute)	20.4 ± 5.9	19.5 ± 7.3	0.823 *
GCS in ED	12.0 ± 4.4	13.1 ± 5.0	0.136 *
AIS of head	1.1 ± 0.5	1.0 ± 0.3	1.000 *
AIS of chest	2.4 ± 1.2	1.7 ± 1.8	0.439 *
AIS of abdomen	2.8 ± 2.0	1.1 ± 1.1	0.016 *
AIS of extremities	1.4 ± 0.9	1.3 ± 1.0	0.662 *
ISS	15.9 ± 12.1	9.8 ± 7.1	0.020 *

Numerical data: mean ± standard deviation. Nominal data: N (percentage). * Student’s *t*-test; ^†^ Chi-square test. Older children = 10 to 17 years old; SBP = systolic blood pressure; ED = emergency department; RR = respiratory rate; GCS = Glasgow Coma Scale; AIS = abbreviated injury scale; ISS = injury severity score.

**Table 2:** Comparisons of demographics, vital signs, injury severities, treatment policies, and outcomes between older children and young adults before and after PSM (*N* = 980).

Torso Trauma Patients (*N* = 321)	Before PSM			After PSM		
	Older Children (*N* = 154)	Young Adults (*N* = 826)	SD *	Older Children (*N* = 92)	Young Adults (*N* = 92)	SD
Male (N)	123 (79.9%)	611 (74.0%)	0.184	70 (76.1%)	72 (78.3%)	0.059
SBP in ED (mmHg)	124.3 ± 71.2	112.2 ± 58.5	0.200	118.4 ± 19.2	119.3 ± 18.0	0.048
Pulse in ED (/min)	101.1 ± 32.2	117.5 ± 41.0	0.413	99.1 ± 22.4	101.5 ± 27.8	0.095
RR in ED (min)	19.0 ± 5.5	22.2 ± 7.1	0.466	20.0 ± 15.5	19.0 ± 19.3	0.057
GCS in ED	13.5 ± 4.4	11.7 ± 2.3	0.527	13.9 ± 4.4	14.0 ± 5.1	0.025
AIS of head	1.1 ± 0.3	1.2 ± 1.6	0.068	1.1 ± 1.1	1.1 ± 1.6	0.000
AIS of chest	2.1 ± 1.8	2.6 ± 1.5	0.322	2.4 ± 2.2	2.2 ± 3.1	0.074
AIS of abdomen	1.9 ± 3.1	2.2 ± 2.0	0.136	2.0 ± 1.6	2.1 ± 1.0	0.075
AIS of extremities	1.0 ± 0.3	1.0 ± 0.5	0.000	1.1 ± 1.0	1.0 ± 1.2	0.091
ISS	14.8 ± 5.6	19.2 ± 7.1	0.639	13.9 ± 11.4	14.0 ± 10.1	0.009
Use of transfusion (N)	71 (46.1%)	501 (60.7%)	0.326	40 (43.5%)	42 (45.7%)	0.049
Torso surgery (N)	55 (35.7%)	277 (33.5)	0.053	30 (32.6%)	30 (32.6%)	0.000
Torso TAE (N)	21 (13.6%)	121 (14.6%)	0.027	8 (8.7%)	9 (9.8%)	0.072
Hospital LOS (day)	9.0 ± 8.3	12.8 ± 11.9	0.358	8.1 ± 3.6	8.0 ± 4.1	0.026
ICU LOS (day)	3.0 ± 3.3	4.5 ± 4.0	0.385	2.6 ± 2.9	2.7 ± 3.1	0.033

Numerical data: mean ± standard deviation, Nominal data: N (percentage). * SD = standardized difference (SD ≥ 0.1 represents significant differences in covariables between groups). Older children = 10 to 17 years old; Young adults = 18 to 35 years old; PSM = propensity score matching; SBP = systolic blood pressure; RR = respiration rate; GCS = Glasgow Coma Scale; ED = emergency department; AIS = abbreviated injury scale; ISS = injury severity score.

**Table 3:** Comparisons of demographics, vital signs, injury severities, treatment policies and outcomes between older children and young adults before and after IPTW (*N* = 980).

Torso Trauma Patients (*N* = 321)	Before IPTW			After IPTW		
	Older Children (*N* = 154)	Young Adults (*N* = 826)	SD *	Older Children	Young Adults	SD
Male (N)	123 (79.9%)	611 (74.0%)	0.184	520 (77.6%)	509 (76.0%)	0.050
SBP in ED (mmHg)	124.3 ± 71.2	112.2 ± 58.5	0.200	122.9 ± 73.1	118.6 ± 44.4	0.071
Pulse in ED (/min)	101.1 ± 32.2	117.5 ± 41.0	0.413	105.0 ± 51.2	107.1 ± 37.7	0.048
RR in ED (min)	19.0 ± 5.5	22.2 ± 7.1	0.466	20.0 ± 11.2	20.0 ± 19.3	0.000
GCS in ED	13.5 ± 4.4	11.7 ± 2.3	0.527	13.0 ± 5.1	12.5 ± 8.6	0.071
AIS of head	1.1 ± 0.3	1.2 ± 1.6	0.068	1.1 ± 0.8	1.1 ± 0.6	0.000
AIS of chest	2.1 ± 1.8	2.6 ± 1.5	0.322	2.3 ± 3.2	2.4 ± 3.3	0.031
AIS of abdomen	1.9 ± 3.1	2.2 ± 2.0	0.136	2.0 ± 1.8	2.1 ± 1.1	0.067
AIS of extremities	1.0 ± 0.3	1.0 ± 0.5	0.000	1.0 ± 0.9	1.0 ± 0.9	0.000
ISS	14.8 ± 5.6	19.2 ± 7.1	0.639	15.2 ± 10.0	16.1 ± 13.3	0.077
Use of transfusion (N)	71 (46.1%)	501 (60.7%)	0.326	357 (53.3%)	549 (52.1%)	0.027
Torso surgery (N)	55 (35.7%)	277 (33.5)	0.053	222 (33.1%)	220 (32.8%)	0.008
Torso TAE (N)	21 (13.6%)	121 (14.6%)	0.027	81 (12.1%)	90 (13.4%)	0.065
Hospital LOS (day)	9.0 ± 8.3	12.8 ± 11.9	0.358	11.4 ± 7.1	12.0 ± 5.8	0.093
ICU LOS (day)	3.0 ± 3.3	4.5 ± 4.0	0.385	3.6 ± 1.9	3.7 ± 2.1	0.050

Numerical data: mean ± standard deviation, Nominal data: N (percentage). * SD = standardized difference (SD ≥ 0.1 represents significant differences in covariables between groups). Older children = 10 to 17 years old, Young adults = 18 to 35 years old. IPTW = inverse probability of treatment weighting, SBP = systolic blood pressure, RR = respiration rate, GCS = Glasgow coma scale, ED = emergency department, AIS = abbreviated injury scale, ISS = injury severity score.

**Table 4:** Comparisons between older children and younger children with torso trauma (*N* = 226).

Variables	Older Children (*N* = 154)	Younger Children (*N* = 72)	*p*-Value
AIS of head	1.1 ± 0.3	0.9 ± 0.5	0.555 *
AIS of chest	2.1 ± 1.8	1.4 ± 0.5	0.013 *
AIS of abdomen	1.9 ± 3.1	0.9 ± 0.8	0.037 *
AIS of extremities	1.0 ± 0.3	2.0 ± 1.1	0.022 *
ISS	14.8 ± 5.6	4.3 ± 10.5	<0.001 *
Torso surgery ^‡^ (N, %)	55 (35.7%)	6 (8.3%)	<0.001 ^†^
TAE (N, %)	21 (13.6%)	3 (4.2%)	0.031 ^†^
Care unit (N, %)			<0.001 ^†^
Pediatric unit	11 (7.1%)	23 (31.9%)	
Adult unit	143 (92.9%)	49 (68.1%)	

Numerical data: mean ± standard deviation. Nominal data: N (percentage). * Student’s t-test; ^†^ Chi-square test. Older children = 10 to 17 years old; AIS = abbreviated injury scale; ISS = injury severity score; TAE = transcatheter arterial embolization. ^‡^ Surgeries were primarily performed by adult trauma surgeons; some patients received postoperative care by pediatric surgeons/pediatricians in the pediatric unit.

## Data Availability

SPSS software package (version 25.0).

## Supplementary Materials

The following supporting information can be downloaded at: https://www.mdpi.com/article/10.3390/children9030444/s1, Figure S1: Distribution of pediatric torso trauma patients who underwent surgery among the age groups; Table S1: International Classification of Diseases, 9th Clinical Modification (ICD-9-CM) Diagnosis Codes (DCODEs) of torso trauma; Table S2: Surgical procedures and indications of pediatric torso trauma patients in different groups; Table S3: Characteristics of young children (age < 10) who underwent surgery.
