# Supplementary material for: Older Children with Torso Trauma Could Be Managed by Adult Trauma Surgeons in Collaboration with Pediatric Surgeons
Source: Children (Basel). 2022 Mar 21;9(3):444. doi: 10.3390/children9030444 (PMC8947374; doi:10.3390/children9030444)
Supplement: Supplementary file 1 [file children-09-00444-s001.zip › children-1635252-supplementary.pdf]

Electronic Supplementary Material Figure S1. Distribution of pediatric torso trauma patients who underwent surgery among the age groups

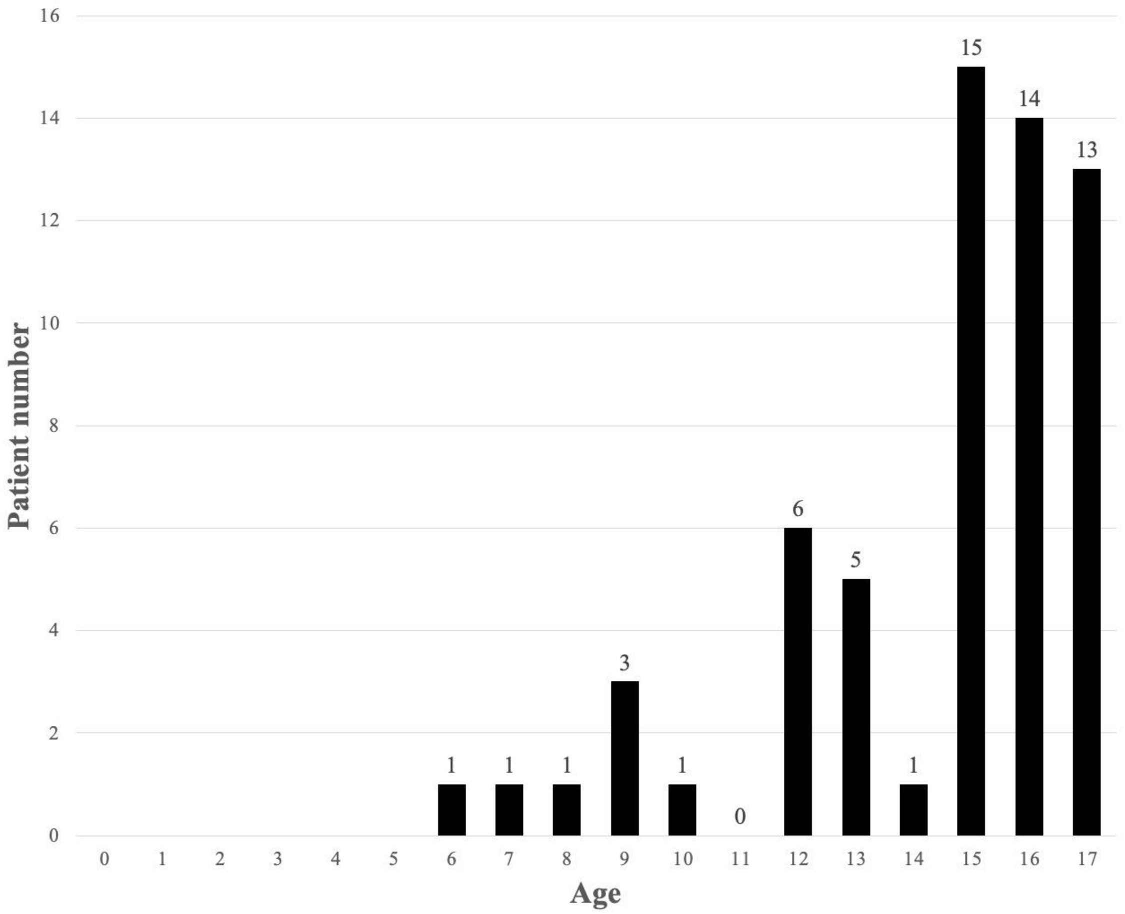

Electronic Supplementary Material Table S1. International Classification of Diseases, 9th Clinical Modification (ICD-9-CM) Diagnosis Codes (DCODEs) of torso trauma

**Torso trauma (860-869)**	**DCODEs of ICD-9-CM**
Chest trauma	
Traumatic pneumothorax and hemothorax	860.0, 860.1, 860.2, 860.3, 860.4, 860.5
Injury to heart and lung	861.00, 861.01, 861.02, 861.03, 861.10, 861.11, 861.12, 861.13, 861.20, 861.21, 861.22, 861.30, 861.31, 861.32
Injury to other and unspecified intrathoracic organs	862.0, 862.1, 862.21, 862.22, 862.29, 862.31, 862.32, 862.39, 862.8, 862.9
Abdominal trauma	

Injury to stomach	863.0, 863.1
Injury to small intestine	863.2-, 863.21, 863.29, 863.30, 863.31, 863.39,
Injury to colon or rectum	863.40, 863.41, 863.42, 863.43, 863.44, 863.45, 863.46, 863.49, 863.50, 863.51, 863.52, 863.53, 863.54, 863.55, 863.56, 863.59,
Injury to other unspecified gastrointestinal sites	863.80, 863.85, 863.89, 863.90, 863.95, 863.99
Injury to liver	864.0, 864.1
Injury to spleen	865.0, 865.1
Injury to kidney	866.0, 866.1
Injury to pancreas	863.81, 863.82, 863.83, 863.84, 863.91, 863.92, 863.93, 863.94
Injury to pelvic organs	867.0, 867.1, 867.2, 867.3, 867.4, 867.5, 867.6, 867.7, 867.8, 867.9
Injury to other intra-abdominal organs	868.0, 868.1
Internal injury to unspecified or ill-defined organ	869.0, 869.1
Fracture of pelvis	808.0, 808.1, 808.2, 808.3, 808.41, 808.42, 808.43, 808.49, 808.51, 808.52, 808.53, 808.59, 808.6, 808.7, 808.8, 808.9

Electronic Supplementary Material Table S2. Surgical procedures and indications of pediatric torso trauma patients in different groups

**Older children (Age: 10-17) (N=55)**	
Surgical procedures	Surgical indications
Splenectomy (N=30, 54.5%)	High grade splenic injury
Hepatorrhaphy (N=6, 10.9%)	High grade liver injury
Hollow viscus injury repair (N=5, 9.0%)	Hollow viscus injury (stomach: 1, small bowel: 3, colon: 1)
Diagnostic laparoscopy (N=5, 9.0%)	Uncertain diagnosis or isolated intra-abdominal free fluid
Exploratory laparotomy (N=4, 7.3%)	Uncertain diagnosis or isolated intra-abdominal free fluid
Mesentery injury repair (N=3, 5.5%)	Mesentery bleeding
Thoracotomy (N=1, 1.8%)	Massive hemothorax
Nephrectomy (N=1, 1.8%)	High grade renal injury with abdominal compartment syndrome
**Younger Children (Age<10) (N=6)**	
Surgical procedures	Surgical indications
Splenectomy (N=3, 50.0%)	High grade splenic injury
Mesentery injury repair (N=1, 16.7%)	Mesentery bleeding
Wound debridement and repair (N=1, 16.7%)	Deep perineal laceration
Diversion colostomy (N=1, 16.7%)	Complicated pelvic fracture

Electronic Supplementary Material Table S3. Characteristics of young children (age < 10) who underwent surgery

Patient	Age	Sex	Body height (cm)	Body weight (kg)	Diagnoses	Surgery
Patient 1	6	F	114	19	Pelvic fracture	Diversion colostomy
Patient 2	8	F	131	40	Perineal laceration	Debridement and primary repair
Patient 3	9	F	138	46	Hight grade splenic injury	Splenectomy
Patient 4	7	M	123	25.2	Hight grade splenic injury	Splenectomy
Patient 5	9	M	140	48	Mesentery injury	Laparotomy for hemostasis
Patient 6	9	M	152	61	Hight grade splenic injury	Splenectomy
